# Public Health Interventions to Improve Antimicrobial Resistance Awareness and Behavioural Change Associated with Antimicrobial Use: A Systematic Review Exploring the Use of Social Media

**DOI:** 10.3390/antibiotics11050669

**Published:** 2022-05-16

**Authors:** Sana Parveen, Nathaly Garzon-Orjuela, Doaa Amin, Patricia McHugh, Akke Vellinga

**Affiliations:** School of Public Health, Physiotherapy & Sports Science, University College Dublin, D04V1W8 Dublin, Ireland; nathaly.garzonorjuela@ucdconnect.ie (N.G.-O.); doaa.amin@ucdconnect.ie (D.A.); patricia.mchugh@nuigalway.ie (P.M.); akke.vellinga@ucd.ie (A.V.)

**Keywords:** antimicrobial resistance, public health intervention, antibiotics, social media, educational campaign, gamification, knowledge and awareness

## Abstract

Introduction: Over the years there have been several interventions targeted at the public to increase their knowledge and awareness about Antimicrobial Resistance (AMR). In this work, we updated a previously published review by Price et al. (2018), on effectiveness of interventions to improve the public’s antimicrobial resistance awareness and behaviours associated with prudent use of antimicrobials to identify which interventions work best in influencing public behaviour. Methods: Five databases—Medline (OVID), CINAHL (EBSCO), Embase, PsycINFO, and Cochrane Central Register of Controlled Trials (CENTRAL-OVID)—were searched for AMR interventions between 2017 and 2021 targeting the public. All studies which had a before and after assessment of the intervention were considered for inclusion. Results: In total, 17 studies were found to be eligible for inclusion in the review. Since there was a variety in the study interventions and in particular outcomes, a narrative synthesis approach was adopted for analysis. Whereas each study showed some impact on awareness and knowledge, none measured long-term impact on behaviours towards antibiotic use, awareness, or knowledge. Engagement was higher in interventions which included interactive elements such as games or videos. Social media was not used for recruitment of participants or as a mode of communication in any AMR interventions included in this review.

## 1. Introduction

Antimicrobial resistance (AMR) has been recognised as one of the greatest threats to human health over the last decade [1,2,3,4,5]. The World Health Organization (WHO) have estimated that antimicrobials add approximately 20 years to life expectancy globally, but this advantage is diminishing due to AMR [6]. In 2019 there were an estimated 4.95 million deaths globally due to AMR [1].

Over the years it has been noticed that a person’s knowledge, attitudes, and beliefs about antimicrobials drives their use. This can be seen in their consultation behaviour by requesting antimicrobials from their GPs or by self-medication [7]. Therefore, one important strategy to control AMR and influence behaviour is appropriate communication about unnecessary antimicrobial use and the spread of AMR [8].

With the growth of social media and its use in every sphere of our lives, it can be expected to have a role in health interventions. Social media for health intervention has been widely used during the COVID-19 pandemic [9,10,11]. A review of the use of social media during the COVID-19 pandemic showed it was the fastest mode of communication for distribution of preventive information and that it can efficiently be used for education, knowledge dissemination, and healthcare awareness [9]. Social media in AMR public health interventions is not well explored and potentially underused.

A systematic review by Price et al. (2018) explored the effectiveness of interventions to improve the public’s awareness of antimicrobial resistance and behaviours associated with prudent use of antimicrobials suggested to use segmentation of the entire population into target groups and devising campaigns for each of these groups [12]. Furthermore, the review showed that school-based interventions and parental educational interventions work best in influencing behavioural change in society [12]. However, the comparison of different public health interventions is challenging due to differences in geographical and economical background as well as different outcomes being measured around the world.

The aforementioned review covered a period from 2000 to 2016 and did not observe any social media aspects as part of these interventions [12]. To explore the impact of social media to improve communication and education in antibiotic consumption and resistance, we aimed to update this review with a particular interest in the use of social media in interventions [12].

## 2. Methods

### 2.1. Search Strategy

The search strategy presented in the supplementary materials of the original review article was adapted to search the various databases—Medline (OVID), CINAHL (EBSCO), Embase, PsycINFO, and Cochrane Central Register of Controlled Trials (CENTRAL-OVID) from 2017 to 2021. The review aimed to replicate the original search strategy as much as possible and search terms were modified to suit each database. The full search strategy is available in Supplementary Materials File S1.

Additional search terms to include the use of social media in public health interventions such as digital marketing, Facebook, LinkedIn, Instagram, Pinterest, TikTok, Twitter, Telegram, and Reddit were added to the intervention keywords during the search. A snowball search through the reference lists of full-text papers was also performed.

Two reviewers (NG and SP) conducted the search on the databases. The search was adapted from the original review to report on the evidence published after the original review and to find out if social media is being used in AMR interventions.

### 2.2. Study Selection

The study selection criteria for study design were similar to the original review which was according to the Cochrane’s Effective Practice and Organization of Core (EPOC) guidelines. However, we also considered study designs with a before and after assessment of the intervention. A time filter was applied and only studies published from 2017 to October 2021 were considered.

Following the PICO criteria for study selection, this review considered all studies that target members of the public. Studies for which participants were recruited from healthcare settings (e.g., patients, hospital staff) were excluded. All interventions designed to increase public awareness and improve antimicrobial stewardship through mass media, social marketing, or printed media campaigns were included with a focus on identifying the use of social media in public health interventions from 2017 onwards, the type of public health interventions conducted, and the messaging used in these public health interventions.

The review considered all studies without any control conditions such as time bound or geographical controls. Main outcomes were all relevant short-, medium-, or long-term outcomes related to AMR and/or antimicrobial stewardship behaviours (knowledge/awareness, learning, public behavioural, and cognition outcomes).

A total of 9435 papers were identified after database screening and 7629 records remained after removing the duplicates. Two reviewers (SP and NG) independently screened the title and abstracts of 7629 records for inclusion. Rayyan software [13,14] was used to screen the title and abstracts by the two reviewers with the blinds turned on.

Conflicts between reviewers were discussed and full texts of such papers were screened. If a consensus could not be reached, a third reviewer was involved to resolve the conflict (AV).

Out of the 9435 records identified, 34 full texts were assessed for eligibility and for inclusion in the study. The full texts of each study were reviewed by two reviewers independently and discussed by all reviewers.

### 2.3. Data Extraction

For data extraction, NG and SP developed a standard tool which was discussed and finalised by all the reviewers. It was agreed it was imperative to extract maximum relevant data to ensure a correct analysis. Table 1 shows the data extraction for all the included studies (detailed version available in Supplementary Materials File S2). All reviewers independently extracted the data for the studies assigned to them and it was crosschecked by a second reviewer.

### 2.4. Quality Assessment

Risk of bias was assessed by three reviewers (S.P., N.G., and D.A.) independently by allocating a subset of included studies to each of the three reviewers. Each study was reviewed by two reviewers independently and the results were crosschecked after. Any conflicts or disagreements in the assessment were resolved by the three reviewers in a discussion meeting. Standard EPOC risk of bias criteria were used for randomised trials, non-randomised trials, and controlled before–after studies. In the previous review, risk of bias was not assessed for Before–After Studies with No Control Group and the risk of bias was assumed to be high for these studies [12]. However, in this review we assessed the risk of bias for Before–After (Pre–Post) studies with no control group using the quality assessment tool developed by NHLBI in 2013 [15]. This was done as the potential biases are likely to be more significant for non-randomised studies than randomised trials, especially if they compare the results between the same group of designs [16].

### 2.5. Data Analysis

The studies included were diverse with respect to the study design, intervention, population, and outcomes. It was not possible to perform further meta-analyses and a narrative synthesis of evidence was adopted.

## 3. Results

For this review, 17 studies were identified and included for evidence synthesis (Table 2). The excluded studies and reasons for exclusion are provided in Supplementary Materials File S3.

The 17 studies included have varied kinds of intervention addressing the public. Intervention types included are educational intervention [3,17,18,19,20,21,22,23,24], theatre intervention [2,25], gamification [26,27], animated film [28], fear-based messaging [29], musical [30], and a digital intervention [31]. Table 1 shows the types of interventions and outcomes from each of these interventions included in this study.

While most of the studies were successful in increasing knowledge of its participants in the short-term (4–6 weeks), long-term impacts of these studies were not evaluated. In one study [28] the authors mentioned that after 6 weeks the impact of the intervention was low and intentions of participants not requesting an antibiotic had waned. In another study [21], it was noted that while the educational intervention helped in increasing the knowledge, there was little impact on the attitudes of participants (public) towards antibiotics.

Interventions targeting school students [3,20,25,30] were impactful and this was also observed in the previous review by Price et al. They reported a substantial increase in knowledge gained and retention of messages between 3 and 6 months after the intervention [3,30].

**Table 1:** Types of interventions and outcomes.

Study	Intervention Type	Outcome
Peer-education as a tool to educate on antibiotics, resistance and use in 16–18-year-olds: A feasibility study in United Kingdom [3]	Educational	Post-intervention students reported a higher median knowledge score.
Effect of didactic educational intervention on improving knowledge of antibiotics use and resistance in Yogyakarta Community [17]	Educational	Post-educational intervention, a large proportion of the respondents (75%) became more aware and mindful about appropriate antibiotic usage. The didactic intervention resulted in higher knowledge and improved practice regarding antibiotics use (*p* < 0.05).
Impact of community-based educational intervention on antibiotic use and resistance awareness among the people living in Ras Al Khaimah, United Arab Emirates [18]	Educational	After the intervention, participants’ knowledge regarding AMR significantly improved—full course of antibiotics should be taken (% change: 50%, *p* < 0.00l), infections from resistant bacteria are difficult to treat (% change: 38%, *p* < 0.001), antibiotics are of no use in viral infections (% change: 72%, *p* < 0.001).
The effect of public health educational campaign regarding antibiotic use and microbial resistance on knowledge, attitude, and practice in the Iran [19]	Educational	After the intervention, the mean of knowledge based on a scale and attitude of the participants was increased (*p* < 0.05).
Using debate to educate young people in schools about antibiotic use and resistance: A before and after evaluation using a questionnaire survey in England [20]	Educational	There was a significant improvement in knowledge after the debate lesson measured through the quantitative questionnaires (*p* < 0.05).
The Consequences of AMR Education and Awareness Raising: Outputs, Outcomes, and Behavioural Impacts of an Antibiotic-Related Educational Activity in Lao PDR [21]	Educational	The intervention had an impact on the awareness levels and understanding about drug resistance, recognition for the term ‘drug resistance’ rose from 27.6% to 91.4% among participants. However, there was little effect on the attitudes of participants.
A mixed-method evaluation of peer-education workshops for school-aged children to teach about antibiotics, microbes and hygiene in England [22]	Educational	The knowledge increase was greater in rural schools compared with schools in urban areas post intervention on the different areas covered in the intervention with (*p* < 0.01) for 2 sections and (*p* < 0.001) for other 2 sections.
A literacy-sensitive approach to improving antibiotic understanding in a community-based [23]	Educational	There was an increase by 2.0 points on the 14-point antibiotic knowledge index score from 11 to 13, (*p* = 0.0011) for the 19 participants completing both the pre and post-test.
Educational intervention to enhance adherence to short-term use of antibiotics in Malta [24]	Educational	The intervention significantly increased adherence to prescribed short-term antibiotics and reduced wastage. Ten percent from intervention group and 24% from control were non-adherent (*p* = < 0.0005), with a 2.8-fold increase in the percentage cost of wasted antibiotics in the control group.
Aston University’s Antimicrobial Resistance (AMR) Roadshow: raising awareness and embedding knowledge of AMR in key stage 4 learners in United Kingdom [25]	Theatre	Post intervention, there was increased accuracy to 91% MCQ questionnaire responses and 100% positive change in response to Likert Scale questions at 12 weeks.
The drugs don’t work: Evaluation of educational theatre to gauge and influence public opinion on antimicrobial resistance in United Kingdom [2]	Theatre	The educational theatre proved to be a successful intervention in increasing the public’s knowledge and understanding about AMR. There was a significant change in scores of participants for 5 out of 8 questions after the play (*p* < 0.05).
The effects of gamification on antimicrobial resistance knowledge and its relationship to dentistry in Saudi Arabia: A randomized controlled trial [26]	Gamification	The intervention had a positive impact on the knowledge gain and retention levels of the participants in the study group (SG) from that of the control group (CG). There was a notable reduction in knowledge in CG compared to SG after a month (t (90.967) = −3.252, *p* = 0.002) as per *t*-test analyses.
Can gaming increase antibiotic awareness in children? A mixed-methods approach in United Kingdom [27]	Gamification	Two areas in the questionnaire showed improvement in knowledge out of seven. These were focused on usage of antibiotics for bacterial infection and the completion of antibiotics course (*p* = 0.01 and *p* < 0.001)
Development and randomized controlled trial of an animated film aimed at reducing behaviours for acquiring antibiotics in United Kingdom [28]	Animated Film	Post intervention, the participants had increased knowledge with the majority of participants (89.1%) agreeing the film was informative though there were not any behavioural changes noted after 6 weeks.
Reducing expectations for antibiotics in primary care: A randomised experiment to test the response to fear-based messages about antimicrobial resistance in United Kingdom [29]	Fear-based messaging	Different levels of fear-based messages yielded different responses; however, the ‘strong fear plus empowerment’ message had a positive impact on participants and they said they were less likely to ask for an antibiotic from their GP (*p* < 0.0001).
‘The Mould that Changed the World’: Quantitative and qualitative evaluation of children’s knowledge and motivation for behavioural change following participation in an antimicrobial resistance musical in United Kingdom [30]	Musical	There was good response recorded at post intervention at two weeks and 6 months with consistency in correct answers with regard to key messaging.
Changing Patient and Public Beliefs About Antimicrobials and Antimicrobial Resistance (AMR) Using a Brief Digital Intervention in United Kingdom [31]	Digital Intervention	Post intervention, there was a significant increase in antibiotic knowledge, AMR concerns, and a reduction in demand for antibiotics based on 17 questions (*p* < 0.0001).

None of the interventions used social media as a tool to reach their participants or convey a message to them. Two interventions [2] and [25] uploaded videos of their interventions on YouTube which shows there is a possibility to reach a bigger audience through social media as well as increase the longevity as videos can be replayed any time.

Intervention showing animated film [28], musical [30] or theatre shows [2] and [25] had a positive impact on the knowledge gained and attitudes of the participants. There was a 4% reduction in intentions to ask for antibiotics in comparison to the control group after watching the animated film [28] which may show the potential of social media for AMR intervention as videos constitute 80% of internet traffic [32].

**Table 2:** Details of Interventions.

Study	Country	Design	Intervention	Outcome Measures	Messaging/Use of Social Media	Results
The drugs don’t work: Evaluation of educational theatre to gauge and influence public opinion on antimicrobial resistance	United Kingdom	Before–After (Pre–Post) Study with No Control Group	Pre-questionnaire to record audience knowledge, attitudes, and opinions on AMR—educational play (presented on three separate occasions at the Birmingham think tank science museum in April 2017 and the Cheltenham science festival in June 2017)—post-questionnaire to record audience knowledge, attitudes, and opinions on AMR.	The audience would record their responses that would reflect their knowledge and understanding of AMR through pre and post questionnaires to the play.	No use of social media; however, the intervention recording is available on YouTube now	An innovative, simple way such as an educational theatre can have a positive impact on the knowledge, understanding, and attitudes towards AMR.
Peer-education as a tool to educate on antibiotics, resistance and use in 16–18-year-olds: A feasibility study	United Kingdom	A feasibility study of a peer-education evaluation using a cluster Randomised Control Trial design	A student-led antibiotic PE sessions in schools for 16–18-year-old biology students, as well as the biology students’ future delivery of these teachings to their 16–18-year-old non-biology peers. It also tested the feasibility of measuring reported student antibiotic use through questionnaires and text messaging.	Students were asked to fill a knowledge questionnaire—pre-knowledge questionnaire, immediately post-knowledge questionnaire, and 3-month follow-up questionnaire.	No use of social media. Text messaged were used	The study demonstrates that cascading the activities from university health students to 16–18-year-old biology students, and then to their non-biology year group, is feasible and may increase antibiotic knowledge.
The effects of gamification on antimicrobial resistance knowledge and its relationship to dentistry in Saudi Arabia: A randomized controlled trial	Saudi Arabia	Single-blinded parallel group randomised controlled trial design	The participants in the study group (SG) received information about AMR by playing a board game aiming to improve AMR knowledge. The participants in the control group (CG) received the same information but by a conventional lecture titled “Antimicrobial Resistance”, which consisted of a Power-Point presentation given by a member of the research team.	The participants were assessed three times: (T1) prior to the intervention, (T2) immediately after the intervention, and (T3) one month later to assess their recall of the information.	Social media was not used	Gamification with a board game can increase AMR knowledge dramatically, with better retention than a traditional lecture. It is a promising way to raise public awareness about AMR and its connection to dentistry.
Development and randomized controlled trial of an animated film aimed at reducing behaviours for acquiring antibiotics	United Kingdom	Randomised Control Trial	Impact of intervention in the form of a short animated video was assessed. The “behavioural change wheel approach” was used to construct the intervention.	Differences in knowledge, attitude/beliefs and intentions between the intervention and control conditions at Time 1 and at Time 2 (6 week follow up) using a questionnaire.	Social media was not used	* Doctor/dentist continued to receive requests for antibiotics. * The animated film showed promise as a tool for preventing patients from requesting antibiotics. * It resulted in a sustained increase in knowledge, but by 6 weeks, the effects on intentions not to ask for antibiotics had faded.
Reducing expectations for antibiotics in primary care: A randomized experiment to test the response to fear-based messages about antimicrobial resistance	United Kingdom	Randomised experiment	To evaluate if fear-based messages with empowering information are more effective than fear based alone.	Online survey measuring participants likelihood to visit a doctor for ILI, request antibiotics, and if antibiotics helped in ILI (influenza-like illness).	Messaging used—‘Antibiotic resistance happens when an antibiotic no longer kills or controls growing bacteria. It is an increasingly serious threat to public health. Without antibiotics that work well, many routine treatments will become increasingly dangerous. Setting broken bones, and even basic operations, rely on access to antibiotics that work. Antibiotic resistance is believed to be caused by unnecessary use of antibiotics, and inappropriate use, such as not taking them as prescribed, skipping doses, or saving them for later use.’	Fear-based messaging can be effective in public campaigns to reduce inappropriate antibiotics use but should be combined with empowering messaging.
‘The Mould that Changed the World’: Quantitative and qualitative evaluation of children’s knowledge and motivation for behavioural change following participation in an antimicrobial resistance musical	United Kingdom	Quantitative study	To enhance children’s knowledge of antibiotic resistance via a musical. Quantitative: online pre-questionnaire then musical workshop intervention then post-questionnaire two weeks after the performance. Qualitative: 29 children randomly picked from each school to participate in a semi-structured focus group before the musical intervention and two weeks after the performance.	Via a quantitative study: the students would answer questions in pre and post questionnaires that reflect their knowledge gain after watching the musical. Via the qualitative study: the students implied better understanding for antimicrobial resistance and the risks of overuse of antibiotics.	Social media was not used	For the quantitative part: 1. knowledge gain was reflected by the children’s ability to answer questions correctly on the post-intervention questionnaire. 2. long term knowledge gain was reflected by a level of correct answers that is consistent between the 2-week and 6-month post-musical questionnaire. For the qualitative part: the children showed better understanding of antimicrobial resistance and the risks of antibiotic overuse and talked about the motivation to decrease the personal use of antibiotic and the influence on friends and family attitudes to antibiotics use.
Changing Patient and Public Beliefs About Antimicrobials and Antimicrobial Resistance (AMR) Using a Brief Digital Intervention	United Kingdom	Pre- and post-intervention study	Participants were given a fictitious case of cold and flu symptoms before being exposed to the intervention. The online intervention included three components: (1) a profiling tool that identified individual beliefs (antibiotic necessity, concerns, and knowledge) that were driving inappropriate antibiotic demand; (2) messages designed to change beliefs and knowledge (i.e., reduce antibiotic necessity and increase antibiotic concerns and knowledge); and (3) an algorithm that linked specific messages to specific beliefs and knowledge.	An online survey containing statements assessing participants’ beliefs about antibiotics and AMR were adapted from the Beliefs about Medicines questionnaire (BMQ), a widely used and validated questionnaire that identifies individual’s beliefs about treatment.	Participants selected via an online survey platform	This is the first study to show that a brief, personalised online intervention can influence patient perceptions about antibiotics and AMR related with inappropriate demand. This has implications for future efforts aimed at reducing antibiotic overuse.
Aston University’s Antimicrobial Resistance (AMR) Roadshow: Raising awareness and embedding knowledge of AMR in key stage 4 learners	United Kingdom	Before and after study without no control group (pre–post)	To develop an interactive collection of workshops at Aston University (Birmingham, UK), which offered Learning Outside the Classroom (LOtC) to Key Stage 4 KS4 ages (14–16) students with the aim of raising awareness and understanding of AMR.	Student questionnaire responses relating to knowledge and understanding of AMR through Likert Scale and Multiple-Choice Type Questions (MCQs) questions over time.	Authors attached video link on YouTube	* The level of pre-existing knowledge and awareness about antibiotics and AMR among the KS4 academy cohort differed significantly. * Findings show that young learners are aware of core AMR issues such as MRSA (Methicillin Resistant Staphylococcus aureus) and the significance of handwashing, but they are less knowledgeable of ‘how’ to correctly wash hands or ‘how’ AMR occurs. Many students believed that ‘people’ become resistant to antibiotics, which is a frequent misunderstanding, and were unaware of the possible utility of natural medicines, such as Manuka Honey, in infection therapy.
Effect of didactic educational intervention on improving knowledge of antibiotics use and resistance in Yogyakarta Community	Indonesia	Quasi-experimental study	To see how effective a pharmacist-led educational intervention is at promoting appropriate antibiotic usage and reducing antibiotic self-medication.	A quantitative study: a total of 268 participants would answer. Pre and 2 weeks post questionnaire that would reflect their knowledge on antibiotics use and resistance.	Social media was not used	The educational intervention, initiated by pharmacists, positively improved the public’s knowledge and practice regarding antibiotics use in Yogyakarta.
Impact of community-based educational intervention on antibiotic use and resistance awareness among the people living in Ras Al Khaimah, United Arab Emirates	Ras Al Khaimah, United Arab Emirates	Pre-test–post-test experimental design	A community-based interventional study was conducted among 100 people. The World Health Organization’s antibiotic resistance: multi-country public awareness survey was used to assess pre-intervention awareness of antibiotics and antibiotic resistance. Following the baseline knowledge assessment, the study participants were provided an educational intervention. After four weeks, the same questionnaire was utilised to assess the impact of the intervention.	To explore the usefulness of a community-based educational intervention in raising awareness about AMR a pre and post questionnaire was given to the participants.	Social media was not used	Antibiotic-related awareness improved dramatically after the intervention. More efforts should be taken to ensure that this increased awareness is translated into long-term health behaviour changes.
The effect of public health educational campaign regarding antibiotic use and microbial resistance on knowledge, attitude, and practice in Iran	Iran	Quasi-experimental study	Antibiotic resistance messaging crafted and designed which included clear and appealing imagery as well as cultural components throughout campaign regions to capture people’s attention. Five specialists were stationed at the sites after collecting data in the target locations (busy places in the city). Two banners with relevant educational material were put in each area’s principal squares. Over 2000 people visited the booths and received training over the course of a week. In public transportation such as buses and subways, posters with eight distinct educational elements were displayed. Furthermore, the campaign’s messages were broadcast on television to people with a variety of tastes and levels of access in various places.	To assess people living in Esfahan’s knowledge, attitudes, and practices about antibiotic resistance, as well as the impact of educational interventions.	Social media was not used; however, traditional mass media marketing channels such as TV were used which helped in reach a wider audience	The main results of this study demonstrated that after the educational campaign, people’s awareness of antibiotic resistance increased.
Using debate to educate young people in schools about antibiotic use and resistance: A before and after evaluation using a questionnaire survey	England	Before and after evaluation using a questionnaire survey	The purpose of this study was to see if the debate kit could improve secondary school students’ (aged 13–16 years) knowledge and understanding of antibiotics and antibiotic resistance.	To assess the increase in knowledge and awareness of antibiotic and antibiotic resistance, students were asked to complete a questionnaire before the lesson began and again immediately after the lesson.	Social media was not used	The findings indicate that the e-Bug antibiotic resistant debate kit can help young people gain a better understanding of antibiotics and antibiotic resistance. Furthermore, kids enjoyed the lesson; thus, this resource should be offered to instructors and schools more extensively.
The Consequences of AMR Education and Awareness Raising: Outputs, Outcomes, and Behavioural Impacts of an Antibiotic-Related Educational Activity in Lao PDR	Thailand	Educational activity assessed through census survey data	To assess a half-day educational activity that interspersed two rounds of complete census surveys, in two peri-urban villages, near a district capital city in Salavan.	To inform the awareness agenda from a social sciences perspective by assessing the outputs, outcomes, and behavioural impacts of an AMR-themed educational activity in the low-income setting through a survey.	Social media was not used	Participants’ awareness and understanding of “drug resistance” were influenced by the educational exercise, although the effects on attitudes were small. The evidence on behavioural effects was limited, but one likely outcome was a disproportionate uptake of antibiotics through professional healthcare practitioners.
Educational intervention to enhance adherence to short-term use of antibiotics	Malta	Randomised controlled trial	To check if a community pharmacist intervention accompanied by an educational leaflet improves adherence and lowers costs in terms of unused antibiotics among patients taking short-term antibiotics in the community, and to see if there is a link between adherence to short-term antibiotics and patients’ general beliefs about medicines.	Patients were asked to count the amount of leftover antibiotic tablets/capsules from the current course, and if any were left, they were termed non-adherent and questioned about why they had leftover antibiotics. Patients were also asked the name and quantity of any leftover antibiotics from past courses that they had at home, as well as the reasons for these unused antibiotics.	Social media was not used	Adherence to prescribed short-term antibiotics has improved dramatically, and waste has decreased as a result of an educational intervention. When distributing medications, telling patients about antibiotic resistance can help them stay on track with their treatment. Taking into account patients’ perceptions is another essential strategy for identifying patients who require additional assistance with antibiotic adherence.
Can Gaming Increase Antibiotic Awareness in Children? A Mixed-Methods Approach	United Kingdom	A mixed methodological approach—questionnaire and focus group	Students aged 9–11 years in primary schools and summer schools in the Bristol and Gloucestershire area played 3 e-Bug games for a total of 15 min. They completed a before and after questionnaire. Further, 48 students took part in 6 focus groups and think-aloud sessions with 4 students were conducted who played the game.	Measuring the change in knowledge after playing the 3 games. Students’ suggestions were also taken to improve the e-Bug games on the website.	Social media was not used	Two games had a significant impact on the knowledge levels of students post intervention. The engagement rate was also higher during the two games.
A mixed-method evolution of peer-education workshops for school- aged children to teach about antibiotics, microbes and hygiene	England	Mixed Methods (Quantitative: before and after + Qualitative: Interviews)	Students attended a science show which consisted of 5 stands covering the topics of microbes, hand hygiene, respiratory hygiene, food hygiene, and antibiotics.	Measurement of knowledge gained about hygiene, the spread of infection, and antibiotics through questionnaire, focus groups, and interviews.	Social media was not used	The study found peer education to be an effective tool for educating students about antibiotics and hygiene.
A literacy-sensitive approach to improving antibiotic understanding in a community-based setting	USA	A pilot pre-test post-test feasibility study	A 30 min live educational seminar about appropriate antibiotic use was showed to the participants. A USD 10 reward was given after completing the pre-test, attending the seminar, and completing the post-test questionnaire, to the participants.	Measurement change in knowledge through a questionnaire.	Social media was not used	The study found the educational seminar to be an effective tool to educate community participants about antibiotics and their role in antibiotic stewardship.

### Quality of Studies

**Figure 1:**
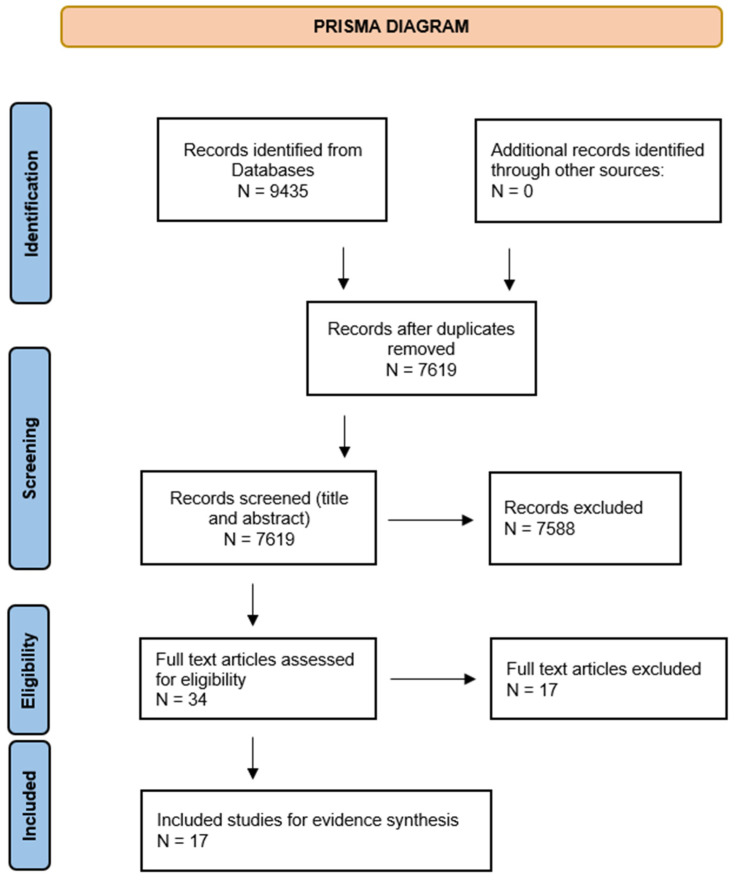
Prisma diagram.

**Figure 2:**
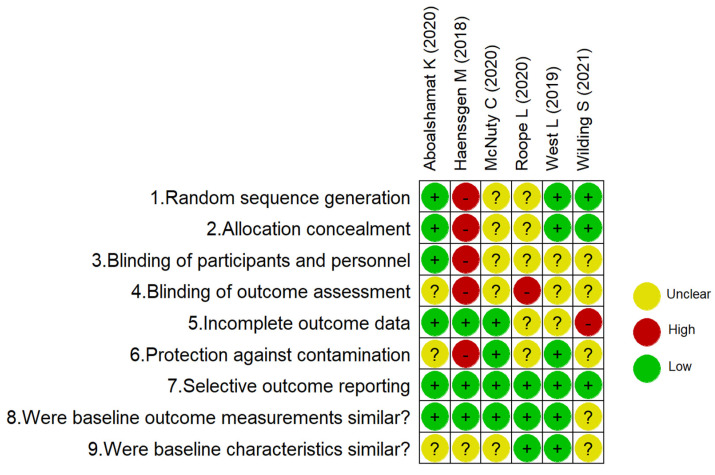
Risk of bias for studies with a separate control group.

The risk of bias of included studies is summarised in Figure 1 and Figure 2 separating controlled studies and Before–After (Pre–Post) Studies with No Control Group. These assessments are described in more detail in Supplementary Materials File S4.

Concerning the risk of bias for studies with a separate control group (Figure 1), three of six studies had a low risk of selection bias due to the generation of a random sequence, with the exception of McNulty et al. (2020) and Roope et al. (2020), where there was insufficient information to allow judgement [3,29]. The study of Haenssgen et al. (2018) was considered to have a high risk of selection bias and protection against contamination being a quasi-experimental design (non-randomised) [21]. Most of the studies that presented an unclear risk for performance and detection bias resulted from the difficulty of blinding, but no description was included in relation to its possible implications [3,21,24,26,28,29].

One study presented a high risk of attrition bias as 13% of data was missing at the second time point [28]. Four studies had insufficient information about the intervention and it was unclear to judge if baseline characteristics were similar [3,26,28] and [21].

**Figure 3:**
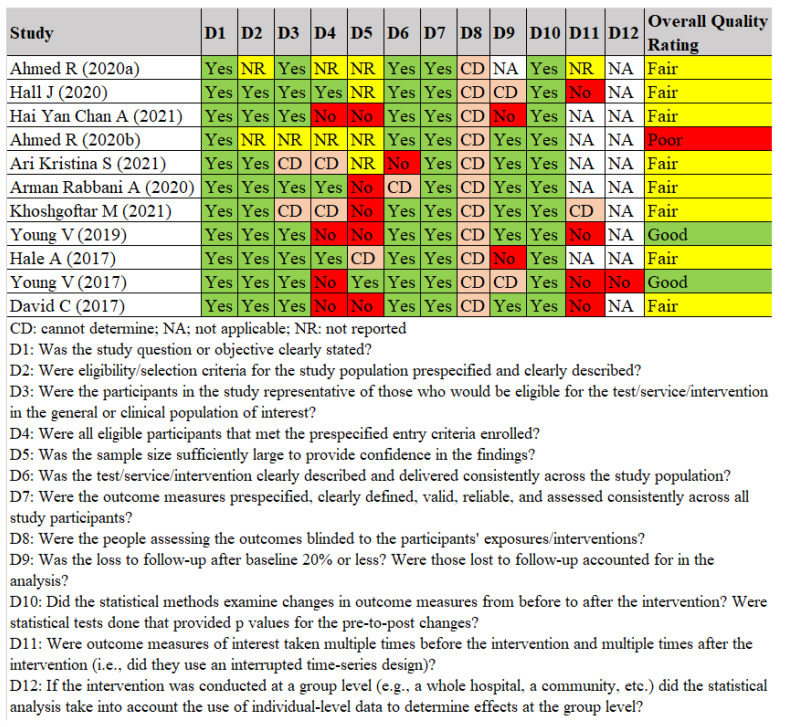
Quality assessment for Before–After (Pre–Post) studies with no control group [2,17,18,19,20,22,23,25,27,30,31].

The quality assessment for Before–After (Pre–Post) studies with no control group are shown in Figure 3. Overall, 7 of 10 studies had a fair quality as not all domains were reported or other information was missing, in particular in relation to the domain on blinding the people assessing the outcomes [2,17,18,19,30,31]. Furthermore, the statistical methods of Ahmed et al. (2020) and Ari et al. (2021) were unclear [17,25].

## 4. Discussion

Each intervention had unique characteristics and structure which makes direct comparison difficult. However, all the interventions included in this review had a positive impact on the level of knowledge gained about AMR and achieved their desired goal among their targeted audience.

There is still widespread absence in understanding, awareness, and general knowledge about antibiotics and their use among the public and interactive, engaging interventions such as theatre plays or videos, have a positive impact on raising public awareness and improving attitudes towards AMR. The scale of each project is, however, reducing the potential impact [2].

Gualano et al. found 50% of the participants had incorrect behaviour towards antimicrobial consumption. The results of their meta-analysis confirmed this finding [33]. To address this challenge, experts in infectious diseases, health communication, and social marketing should be an integral part of antimicrobial awareness campaign planning teams [8].

An interesting aspect highlighted by Ahmed et al. [2] is that important messages are often ‘pushed’ onto the audience in public health interventions, and that in interactive play format, concerns are presented and the audience is challenged to examine their understanding. The audience gets ‘drawn’ into the important concepts in this way, giving them a deeper sense of ownership, involvement, and engagement with the subject.

The success of an intervention which used gamification was presented by Aboalshamat et al., who found not only an immediate but also long-term retention if the game was engaging. This can be explained by Csikszentmihalyi’s flow theory which states that when a person is immersed in an event, they are more likely to be fully engrossed and focused on the work at hand [26].

Overall, the use of social media is absent in the delivery of interventions to improve AMR awareness. Although, it was shown that engagement seems to be the key in gathering attention as well as having long-term impact. Social media is considered one of the main tools in current society to get engagement. In 2021, a global record 4.5 billion active social media users were observed, a 13 % year on year increase [34].

Studies on social media during the pandemic found beneficial connections between the components of a social media campaign, public health awareness, and behavioural change amid COVID-19 [9,10,11]. However, different countries have different favourite social media platforms, use different types of messages, and vary in source sender types [11]. While social media has clear advantages, it can also quickly spread false information [10]. For instance, false information was being tweeted far more than correct information even though it had a lower engagement rate or retweets while scientifically correct tweets had higher retweets and engagement [10].

Social media use during the pandemic shows its potential for AMR information campaigns and provides lessons. One of which is that clinicians and health experts/researchers should serve as the source of correct information [35]. Additionally, appropriate social media platforms should be used depending on the target audience—the younger generation would prefer Instagram or TikTok while the older generations would prefer Facebook [36].

Additionally, a common measurement tool such as a questionnaire needs to be developed to standardise the measure the effect in each intervention. Currently, the lack of such a common outcome measurement tool makes it difficult to compare these interventions against one another and find out what works best in educating the public and truly influencing behaviour change.

### Limitations

Our study aimed to identify the interventions to educate the public about AMR and we had to adapt a narrative synthesis approach as each intervention had different outcome and methods of evaluation, due to which a meta-analysis was not possible. This made it impossible to evaluate the interventions against each other directly by using a statistical analysis. Additionally, no intervention measured long-term outcomes, beyond 6 months, and it was therefore not possible to confirm if there was any real long-term retention of knowledge in any intervention.

## 5. Conclusions

As seen from the different interventions included in this review, the long-term impact of any intervention is not evaluated. While most of the interventions have an impact on knowledge gained immediately after the intervention, a shift in attitudes towards sustained knowledge on AMR and use of antibiotics is lacking. The mode of engagement and its role in long-term retention needs to be considered and future interventions can take learnings from the pandemic social media campaigns.

## Data Availability

Not applicable.

## Supplementary Materials

The following supporting information can be downloaded at: https://www.mdpi.com/article/10.3390/antibiotics11050669/s1, File S1: Evidence search report in electronic databases, File S2: Data extraction, File S3: List of studies excluded and reasons for their exclusion—full text, File S4: Risk of bias; File S5: Revised Search Strategy to check for Telegram and Reddit.

## References

[1] Murray C.J., Ikuta K.S., Sharara F., Swetschinski L., Aguilar G.R., Gray A., Han C., Bisignano C., Rao P., Wool E. (2022). Global burden of bacterial antimicrobial resistance in 2019: A systematic analysis. Lancet.

[2] Ahmed R., Bashir A., Brown J., Cox J., Hilton A., Hilton C., Lambert P., Theodosiou E., Tritter J., Watkin S. (2020). The drugs don’t work: Evaluation of educational theatre to gauge and influence public opinion on antimicrobial resistance. J. Hosp. Infect..

[3] McNulty C.A.M., Syeda R.B., Brown C.L., Bennett C.V., Schofield B., Allison D.G., Verlander N.Q., Francis N. (2020). Peer-education as a tool to educate on antibiotics, resistance and use in 16–18-year-olds: A feasibility study. Antibiotics.

[4] WHO (2014). Antimicrobial Resistance: Global Report on Surveillance.

[5] WHO (2015). Global action plan on antimicrobial resistance. Global Action Plan on Antimicrobial Resistance.

[6] National Institute for Health and Care Excellence (2017). NICE Guideline: Antimicrobial Stewardship: Changing Risk-Related Behaviours in the General Population. https://www.nice.org.uk/guidance/ng63/resources/antimicrobial-stewardship-changing-riskrelated-behaviours-in-the-general-population-pdf-1837572082117.

[7] McNulty C.A., Nichols T., French D.P., Joshi P., Butler C.C. (2013). Expectations for consultations and antibiotics for respiratory tract infection in primary care: The RTI clinical iceberg. Br. J. Gen. Pract..

[8] Huttner B., Saam M., Moja L., Mah K., Sprenger M., Harbarth S., Magrini N. (2019). How to improve antibiotic awareness campaigns: Findings of a WHO global survey. BMJ Glob. Health.

[9] Verma B. (2021). Social Media Analysis during Covid-19: A Systematic Review. Int. J. Recent Technol. Eng. (IJRTE).

[10] Tsao S.-F., Chen H., Tisseverasinghe T., Yang Y., Li L., Butt Z.A. (2021). What social media told us in the time of COVID-19: A scoping review. Lancet Digit. Health.

[11] Abuhashesh M.Y., Al-Dmour H., Masa’Deh R., Salman A., Al-Dmour R., Boguszewicz-Kreft M., AlAmaireh Q.N. (2021). The Role of Social Media in Raising Public Health Awareness during the Pandemic COVID-19: An International Comparative Study. Informatics.

[12] Price L., Gozdzielewska L., Young M., Smith F., Macdonald J., McParland J., Williams L., Langdridge D., Davis M.D.M., Flowers P. (2018). Effectiveness of interventions to improve the public’s antimicrobial resistance awareness and behaviours associated with prudent use of antimicrobials: A systematic review. J. Antimicrob. Chemother..

[13] Kellermeyer L., Harnke B., Knight S. (2018). Covidence and Rayyan. J. Med. Libr. Assoc..

[14] Ouzzani M., Hammady H., Fedorowicz Z., Elmagarmid A. (2016). Rayyan—A web and mobile app for systematic reviews. Syst. Rev..

[15] Nhlbi N. (2022). Study Quality Assessment Tools National Heart, Lung and Blood Institute. https://www.nhlbi.nih.gov/health-topics/study-quality-assessment-tools.

[16] Reeves B.C., Deeks J.J., Higgins J.P.T., Shea B., Tugwell P., Wells G.A., Higgins J.P.T., Thomas J., Chandler J., Cumpston M., Li T., Page M.J., Welch V.A., the Cochrane Non-Randomized Studies of Interventions Methods Group (2019). Chapter 24: Including non-randomized studies on intervention effects. Cochrane Handbook for Systematic Reviews of Interventions.

[17] Kristina S.A., Salsabila N.N., Yulianto Y., Fortwengel G. (2021). Effect of didactic educational intervention on improving knowledge of antibiotics use and resistance in Yogyakarta Community. Pharm. Sci. Asia.

[18] Rabbani S.A., Sridhar S.B., Abazer D., Ahmed H.S., Usman H.A., Mahtab A., El-Dahiyat F. (2020). Impact of community-based educational intervention on antibiotic use and resistance awareness among the people living in Ras Al Khaimah, United Arab Emirates. J. Pharm. Health Serv. Res..

[19] Pirzadeh A., Khoshgoftar M., Zamani-Alavijeh F., Kasaian N., Shahzamani K., Rostami S., Nakhodian Z. (2021). The effect of public health educational campaign regarding antibiotic use and microbial resistance on knowledge, attitude, and practice in the Iran. J. Educ. Health Promot..

[20] Young V.L., Berry M., Verlander N.Q., Ridgway A., McNulty C.A. (2019). Using debate to educate young people in schools about antibiotic use and resistance: A before and after evaluation using a questionnaire survey. J. Infect. Prev..

[21] Haenssgen M.J., Xayavong T., Charoenboon N., Warapikuptanun P., Zaw Y.K. (2018). The Consequences of AMR Education and Awareness Raising: Outputs, Outcomes, and Behavioural Impacts of an Antibiotic-Related Educational Activity in Lao PDR. Antibiotics.

[22] Young V.L., Cole A., Lecky D.M., Fettis D., Pritchard B., Verlander N.Q., Eley C.V., McNulty C.A.M. (2017). A mixed-method evaluation of peer-education workshops for school-aged children to teach about antibiotics, microbes and hygiene. J. Antimicrob. Chemother..

[23] David C.M., O’Neal K.S., Miller M.J., Johnson J.L., Lloyd A.E. (2017). A literacy-sensitive approach to improving antibiotic understanding in a community-based setting. Int. J. Pharm. Pract..

[24] West L.M., Cordina M. (2019). Educational intervention to enhance adherence to short-term use of antibiotics. Res. Soc. Adm. Pharm..

[25] Ahmed R., Bashir A., Brown J.E., Cox J.A., Hilton A.C., Jordan S.L., Theodosiou E., Worthington T. (2020). Aston University’s Antimicrobial Resistance (AMR) Roadshow: Raising awareness and embedding knowledge of AMR in key stage 4 learners. Infect. Prev. Pract..

[26] Aboalshamat K., Khayat A., Halwani R., Bitan A., Alansari R. (2020). The effects of gamification on antimicrobial resistance knowledge and its relationship to dentistry in Saudi Arabia: A randomized controlled trial. BMC Public Health.

[27] Hale A.R., Young V.L., Grand A., McNulty C.A.M., De Quincey E., Ridgway A., Dockterman D. (2017). Can gaming increase antibiotic awareness in children? a mixed-methods approach. JMIR Serious Games.

[28] Wilding S., Kettu V., Thompson W., Howard P., Jeuken L.J.C., Pownall M., Conner M., Sandoe J.A.T. (2021). Development and randomized controlled trial of an animated film aimed at reducing behaviours for acquiring antibiotics. JAC-Antimicrob. Resist..

[29] Roope L.S.J., Herd N.M.S., Pouwels K.B., Castro-Sanchez E., Sallis A., Hopkins S., Robotham J.V., Crook D.W., Peto T., Peters M. (2020). Reducing expectations for antibiotics in primary care: A randomised experiment to test the response to fear-based messages about antimicrobial resistance. BMC Med..

[30] Hall J., Jones L., Robertson G., Hiley R., Nathwani D., Perry M.R. (2020). ‘The Mould that Changed the World’: Quantitative and qualitative evaluation of children’s knowledge and motivation for behavioural change following participation in an antimicrobial resistance musical. PLoS ONE.

[31] Chan A.H.Y., Horne R., Lycett H., Raebel E., Guitart J., Wildman E., Ang K. (2021). Changing Patient and Public Beliefs About Antimicrobials and Antimicrobial Resistance (AMR) Using a Brief Digital Intervention. Front. Pharmacol..

[32] Video L. (2021). 80% of the Internet Is Video—Are You Using It Effectively?. https://wwwlaivideocom/blog/80-internet-video-are-you-using-it-effectively.

[33] Gualano M.R., Gili R., Scaioli G., Bert F., Siliquini R. (2015). General population’s knowledge and attitudes about antibiotics: A systematic review and meta-analysis. Pharmacoepidemiol. Drug Saf..

[34] Dean B. (2021). Social Network Usage & Growth Statistics: How Many People Use Social Media in 2022?. https://backlinko.com/social-media-users.

[35] Malecki K.M.C., Keating J.A., Safdar N. (2020). Crisis Communication and Public Perception of COVID-19 Risk in the Era of Social Media. Clin. Infect. Dis..

[36] Auxier B., Anderson M. (2021). Social Media Use in 2021. Pew Res. Cent..

