# Supplementary material for: Public Health Interventions to Improve Antimicrobial Resistance Awareness and Behavioural Change Associated with Antimicrobial Use: A Systematic Review Exploring the Use of Social Media
Source: Antibiotics (Basel). 2022 May 16;11(5):669. doi: 10.3390/antibiotics11050669 (PMC9137793; doi:10.3390/antibiotics11050669)
Supplement: Supplementary file 1 [file antibiotics-11-00669-s001.zip › File S1 - Electronic search report..pdf]

File S1. Evidence search report in electronic databases

Electronic search report No. 1	
**Electronic database**	MEDLINE(R) ALL 1946 to 27 April, 2022
**Platform**	OVID
**Date of search**	27-04-2022
**Restriction of language**	None
**Other limits**	None
**Search strategy (results)**	1 (superbug* or superinfection*).ti,ab. 6574<br>2 *Superinfection/ 1202<br>3 (multiresistan* or multi-resistan* or MDR or MDRO or AMR).ti,ab. 38866<br>4 ((drug* or multidrug or multi-drug or antimicrobial* or anti-microbial* or antiinfection* or anti-infection* or antibacterial* or anti-bacterial* or antibiotic* or anti-biotic*) adj4 (resistan* or toleran*)).ti,ab. 245122<br>5 *Drug Resistance/ 11448<br>6 *Drug Resistance, Microbial/ 15381<br>7 *Drug Resistance, Multiple/ 7520<br>8 *Drug Tolerance/ 4156<br>9 (r factor* or resistant factor*).ti,ab. 4007<br>10 *R Factors/ 2780<br>11 ((antimicrobial or anti-microbial or antiinfection or anti-infection or antibacterial or anti-bacterial or antibiotic or anti-biotic) adj4 stewardship).ti,ab. 6902<br>12 *Anti-Infective Agents/ 42767<br>13 *Anti-Bacterial Agents/ 230652<br>14 1 or 2 or 3 or 4 or 5 or 6 or 7 or 8 or 9 or 10 or 11 or 12 or 13 507496<br>15 (public or population or community or citizen* or household* or resident* or consumer* or carer* or caregiver* or people or adult* or men or women or mother* or father* or parent* or student* or pupil* or children or adolescent* or teenager*).ti,ab. 6313316<br>16 *Caregivers/ 30081<br>17 *Men/ 2310<br>18 *Women/ 11642<br>19 *Parents/ 38773<br>20 *Fathers/ 5573<br>21 *Mothers/ 30706<br>22 *Students/ 38353<br>23 15 or 16 or 17 or 18 or 19 or 20 or 21 or 22 6328222<br>24 *Program Evaluation/ 11469<br>25 ((behavio#r adj3 (change* or changing or alter* or modification* or modify or modifying or modifies or modified)) or intention*).ti,ab. 114721<br>26 *Motivation/ 31549<br>27 *Intention/ 6774<br>28 (attitude* or belief* or view* or opinion* or expectation* or anticipat* regret or knowledge or awareness or health literacy or risk perception* or perceived risk* or perceived susceptibility or illness perception* or treatment perception*).ti,ab. 1752751<br>29 *Attitude to Health/ 43877<br>30 *Health Knowledge, Attitudes, Practice/ 67995<br>31 *Health Literacy/ 6108

	32 (perceived behavio#ral control or autonomy or capacity or self-efficacy or competen* or confidence or ability or skill*).ti,ab. 2359205<br>33 *Self Efficacy/ 9486<br>34 (perceived norm* or injunctive norm* or subjective norm* or descriptive norm* or moral norm* or self-identity).ti,ab. 5189<br>35 *Social Norms/ 1043<br>36 ((medication* or drug* or antimicrobial* or anti-microbial* or antiinfection* or anti-infection* or antibacterial* or anti-bacterial* or antibiotic* or anti-biotic*) adj4 (use* or consumption or purchas* or dispos* or storage or storing or sharing or shared or expect* or request*)).ti,ab. 355693<br>37 ((inappropriat* or irrational or imprudent or unnecessar* or irresponsibl* or misuse* or improper* or mistake* or indiscriminat* or suboptimum* or bad or overuse* or excessiv* or vary or varied or variation or poor or unsafe*) adj4 (use* or utili?e or treatment or consumption or purchas* or dispos* or storage or sharing or shared or expectation* or request*)).ti,ab. 98333<br>38 ((appropriat* or rational or prudent or judicious* or optimal* or correct or proper* or responsibl* or safe* or good or decreas* or limit* or curb* or minimi?e* or minimal* or lessen* or curtail* or abate* or restrict* or lower* or discontinue* or delay*) adj4 (use* or utili?e or treatment or consumption or purchas* or dispos or storage or sharing or shared or expectation* or request*)).ti,ab. 721908<br>39 *Drug-Seeking Behavior/ 1094<br>40 (compliance or concordance or adherence).ti,ab. 306305<br>41 *Medication Adherence/ 13883<br>42 (self-car* or self-manag* or self-monitor* or self-medicat*).ti,ab. 54554<br>43 *Self Care/ 19294<br>44 *Self Medication/2335<br>45 44 or 45 or 46 or 47 or 48 or 49 or 50 or 51 or 52 or 53 or 54 or 55 or 56 or 57 or 58 or 59 or 60 or 61 or 62 or 63 or 64 5109261<br>46 **Telegram.ti,ab. 130**<br>**47 reddit.ti,ab. 321**<br>48 46 or 47 451<br>49 14 and 23 and 45 and 48 0
**Number of references found**	0
**Electronic search report No. 2**	
**Electronic database**	Embase
**Platform**	Elsevier
**Date of search**	27-04-2022
**Restriction of language**	None
**Other limits**	None
**Search strategy (results)**	1 superbug*:ti,ab OR superinfection*:ti,ab (8714)<br>2 'superinfection'/mj (1970)<br>3 multiresistan*:ti,ab OR 'multi resistan*':ti,ab OR mdr:ti,ab OR mdro:ti,ab OR amr:ti,ab (54977)<br>4 ((drug* OR multidrug OR 'multi-drug' OR antimicrobial* OR 'anti-microbial*' OR antiinfection* OR 'anti-infection*' OR antibacterial* OR 'anti-bacterial*' OR antibiotic* OR 'anti-biotic*') NEXT/4 (resistan* OR toleran*)):ti,ab (284232)<br>5 'drug resistance'/mj (32388)

	6 'antibiotic resistance'/mj (67487)<br>7 'multidrug resistance'/mj (17941)<br>8 'drug tolerance'/mj (11084)<br>9 'r factor*':ti,ab OR 'resistant factor*':ti,ab (4007)<br>10 'r factor'/mj (2891)<br>11 ((antimicrobial OR 'anti-microbial' OR antiinfection OR 'anti-infection' OR antibacterial OR 'anti-bacterial' OR antibiotic OR 'anti-biotic') NEXT/4 stewardship):ti,ab (11054)<br>12 'antiinfective agent'/mj (106,926)<br>13 'antibiotic therapy'/mj (19,049)<br>14 #1 OR #2 OR #3 OR #4 OR #5 OR #6 OR #7 OR #8 OR #9 OR #10 OR #11 OR #12 OR #13 (487,646)<br>15 public:ti,ab OR population:ti,ab OR community:ti,ab OR citizen*:ti,ab OR household*:ti,ab OR resident*:ti,ab OR consumer*:ti,ab OR carer*:ti,ab OR caregiver*:ti,ab OR people:ti,ab OR adult*:ti,ab OR men:ti,ab OR women:ti,ab OR mother*:ti,ab OR father*:ti,ab OR parent*:ti,ab OR student*:ti,ab OR pupil*:ti,ab OR children:ti,ab OR adolescent*:ti,ab OR teenager*:ti,ab (8,603,296)<br>16 'caregiver'/mj (26,400)<br>17 'male'/mj (49,402)<br>18 'female'/mj (96,829)<br>19 'parent'/mj (22,082)<br>20 'father'/mj (4213)<br>21 'mother'/mj (22,419)<br>22 'student'/mj (29,442)<br>23 #15 OR #16 OR #17 OR #18 OR #19 OR #20 OR #21 OR #22 (8,651,809)<br>24 **telegram:ti,ab OR reddit:ti,ab (645)**<br>25 'program evaluation'/mj (2572)<br>26 ((behavio$r NEXT/3 (change* OR changing OR alter* OR modification* OR modify OR modifying OR modifies OR modified)):ti,ab) OR intention*:ti,ab (168,030)<br>27 'motivation'/mj (33,095)<br>28 'behavior'/mj (70,764)<br>29 attitude*:ti,ab OR belief*:ti,ab OR view*:ti,ab OR opinion*:ti,ab OR expectation*:ti,ab OR 'anticipat* regret':ti,ab OR knowledge:ti,ab OR awareness:ti,ab OR 'health literacy':ti,ab OR 'risk perception*':ti,ab OR 'perceived risk*':ti,ab OR 'perceived susceptibility':ti,ab OR 'illness perception*':ti,ab OR 'treatment perception*':ti,ab (2,275,676)<br>30 'attitude to health'/mj (62,595)<br>31 'health literacy'/mj (6801)<br>32 'perceived behavio$ral control':ti,ab OR autonomy:ti,ab OR capacity:ti,ab OR 'self-efficacy':ti,ab OR competen*:ti,ab OR confidence:ti,ab OR ability:ti,ab OR skill*:ti,ab (2,980,962)<br>33 'self concept'/mj (32,649)<br>34 'perceived norm*':ti,ab OR 'injunctive norm*':ti,ab OR 'subjective norm*':ti,ab OR 'descriptive norm*':ti,ab OR 'moral norm*':ti,ab OR 'self-identity':ti,ab (6131)<br>35 'social norm'/mj (1179)<br>36 ((medication* OR drug* OR antimicrobial* OR 'anti-microbial*' OR antiinfection* OR 'anti-infection*' OR antibacterial* OR 'anti-bacterial*' OR antibiotic* OR 'anti-biotic*') NEXT/4 (use* OR

	consumption OR purchas* OR dispos* OR storage OR storing OR sharing OR shared OR expect* OR request*)):ti,ab (313,796)<br>37 ((inappropriat* OR irrational OR imprudent OR unnecessar* OR irresponsibl* OR misuse* OR improper* OR mistake* OR indiscriminat* OR suboptimum* OR bad OR overuse* OR excessiv* OR vary OR varied OR variation OR poor OR unsafe*) NEXT/4 (use* OR utili?e OR treatment OR consumption OR purchas* OR dispos* OR storage OR sharing OR shared OR expectation* OR request*)):ti,ab (91,427)<br>38 ((appropriat* OR rational OR prudent OR judicious* OR optimal* OR correct OR proper* OR responsibl* OR safe* OR good OR decreas* OR limit* OR curb* OR minimi?e* OR minimal* OR lessen* OR curtail* OR abate* OR restrict* OR lower* OR discontinue* OR delay*) NEXT/4 (use* OR utili?e OR treatment OR consumption OR purchas* OR dispos OR storage OR sharing OR shared OR expectation* OR request*)):ti,ab (662,806)<br>39 'drug seeking behavior'/mj (962)<br>40 compliance:ti,ab OR concordance:ti,ab OR adherence:ti,ab (482,897)<br>41 'medication compliance'/mj (11,595)<br>42 'self-car*':ti,ab OR 'self-manag*':ti,ab OR 'self-monitor*':ti,ab OR 'self-medicat*':ti,ab (80,425)<br>43 'self care'/mj (21.124)<br>44 'self medication'/mj (4142)<br>45 #25 OR #26 OR #27 OR #28 OR #29 OR #30 OR #31 OR #32 OR #33 OR #34 OR #35 OR #36 OR #37 OR #38 OR #39 OR #40 OR #41 OR #42 OR #43 OR #44 (6,315,153)<br>46 #14 AND #23 AND #24 AND #45 (0)
**Number of references found**	0
**Electronic search report No. 3**	
**Electronic database**	Cochrane Central Register of Controlled Trials September 2021
**Platform**	OVID
**Date of search**	27-04-2022
**Restriction of language**	None
**Other limits**	None
**Search strategy (results)**	1 (superbug* or superinfection*).ti,ab. 438<br>2 (multiresistan* or multi-resistan* or MDR or MDRO or AMR).ti,ab. 1404<br>3 ((drug* or multidrug or multi-drug or antimicrobial* or anti-microbial* or antiinfection* or anti-infection* or antibacterial* or anti-bacterial* or antibiotic* or anti-biotic*) adj4 (resistan* or toleran*)).ti,ab. 10033<br>4 Drug Resistance/ 1655<br>5 Drug Resistance, Microbial/ 776<br>6 Drug Resistance, Multiple/ 151<br>7 Drug Tolerance/ 1976<br>8 (r factor* or resistant factor*).ti,ab. 8

	9 ((antimicrobial or anti-microbial or antiinfection or anti-infection or antibacterial or anti-bacterial or antibiotic or anti-biotic) adj4 stewardship).ti,ab. 443<br>10 Anti-Infective Agents/ 2741<br>11 Anti-Bacterial Agents/ 11411<br>12 1 or 2 or 3 or 4 or 5 or 6 or 7 or 8 or 9 or 10 or 11 26330<br>13 (public or population or community or citizen* or household* or resident* or consumer* or carer* or caregiver* or people or adult* or men or women or mother* or father* or parent* or student* or pupil* or children or adolescent* or teenager*).ti,ab. 676087<br>14 Caregivers/ 2530<br>15 Men/ 65<br>16 women/ 251<br>17 Parents/ 3642<br>18 Fathers/ 219<br>19 Mothers/ 2150<br>20 Students/ 3293<br>21 13 or 14 or 15 or 16 or 17 or 18 or 19 or 20 676626<br>22 **(telegram or reddit).ti,ab. 104**<br>23 12 and 21 and 22 0
**Number of references found**	0
	**Electronic search report No. 4**
**Electronic database**	CINAHL Complete
**Platform**	EBSCOhost
**Date of search**	27-04-2022
**Restriction of language**	None
**Other limits**	None
**Search strategy (results)**	S1 TX superbug* OR TX superinfection (3548)<br>S2 MH Superinfection (229)<br>S3 TX multiresistan* OR TX "multi-resistan*" OR AB MDR OR AB MDRO OR AB AMR (6226)<br>S4 TX ((drug* OR multidrug OR "multi-drug" OR antimicrobial* OR "anti-microbial*" OR antiinfection* OR "anti-infection*" OR antibacterial* OR "anti-bacterial*" OR antibiotic* OR "anti-biotic*") N4 (resistant OR resistance OR tolerant OR tolerance)) (88,334)<br>S5 MH "Drug Resistance" OR MH "Drug Resistance, Microbial" OR MH "Drug Resistance, Multiple" OR MH "Drug Tolerance" OR MH "antibiotic resistance" OR MH "multidrug resistance" (35,063)<br>S6 TX "r factor*" OR TX "resistant factor*" (1251)<br>S7 TX ((antimicrobial OR "anti-microbial" OR antiinfection OR "anti-infection" OR antibacterial OR "anti-bacterial" OR antibiotic OR "anti-biotic") N4 (stewardship)) (5958)<br>S8 TX "Anti-Infective Agents" (441)<br>S9 TX "Anti-Bacterial Agents" (160)<br>S10 (S1 OR S2 OR S3 OR S4 OR S5 OR S6 OR S7 OR S8 OR S9) (96,099)<br>S11 AB (public OR population OR community OR citizen* OR household* OR resident* OR consumer* OR carer* OR caregiver* OR

	people OR adult* OR men OR women OR mother* OR father* OR parent* OR student* OR pupil* OR children OR adolescent* OR teenager*) (1,947,026)<br>S12 MH Caregivers (40,170)<br>S13 MH Men (6053)<br>S14 MH Women (22,467)<br>S15 MH Parents (48,949)<br>S16 MH Fathers (7286)<br>S17 MH Mothers (34,635)<br>S18 MH Students (18,893)<br>S19 (S11 OR S12 OR S13 OR S14 OR S15 OR S16 OR S17 OR S18) (2,003,527)<br>**S20 AB (telegram OR reddit) (234)**<br>S21 MH “Program Evaluation” (45,978)<br>S22 TX ((behavio#r N3 (change* OR changing OR alter* OR modification* OR modify OR modifying OR modifies OR modified)) OR intention*) (215,472)<br>S23 MH "Motivation" (44,027)<br>S24 MH "Intention" (9259)<br>S25 AB (attitude* OR belief* OR view* OR opinion* OR expectation* OR "anticipat* regret" OR knowledge OR awareness OR "health literacy" OR "risk perception*" OR "perceived risk*" OR "perceived susceptibility" OR "illness perception*" OR "treatment perception*") (529,234)<br>S26 MH "Attitude to Health" (47,870)<br>S27 TX "Health Knowledge, Attitudes, Practice" (243)<br>S28 MH "Health Literacy" (5475)<br>S30 AB (“perceived behavio#ral control” OR autonomy OR capacity OR "self-efficacy" OR competen* OR confidence OR ability OR skill*) (586,649)<br>S31 TX "Self Efficacy" (62,806)<br>S32 TX ("perceived norm*" OR "injunctive norm*" OR "subjective norm*" OR "descriptive norm*" OR "moral norm*" OR "self-identity") (10,673)<br>S33 MH "Social Norms" (2318)<br>S34 AB ((medication* OR drug* OR antimicrobial* OR "anti-microbial*" OR antiinfection* OR "anti-infection*" OR antibacterial* OR "anti-bacterial*" OR antibiotic* OR "anti-biotic*") N4 (use OR used OR usage OR consumption OR purchas* OR dispos* OR storage OR storing OR sharing OR shared OR expect* OR request*)) (92,257)<br>S35 AB ((inappropriat* OR irrational OR imprudent OR unnecessar* OR irresponsibl* OR misuse* OR improper* OR mistake* OR indiscriminat* OR suboptimum* OR bad OR overuse* OR excessiv* OR vary OR varied OR variation OR poor OR unsafe*) N4 (use OR usage OR utili?e OR treatment OR consumption OR purchas* OR dispos* OR storage OR sharing OR shared OR expectation* OR request*)) (31,910)<br>S36 AB ((appropriat* OR rational OR prudent OR judicious* OR optimal* OR correct OR proper* OR responsibl* OR safe* OR good OR decreas* OR limit* OR curb* OR minimi?e* OR minimal* OR lessen* OR curtail* OR abate* OR restrict* OR lower* OR discontinue* OR delay*) N4 (use OR usage OR utili?e OR treatment OR consumption OR purchas* OR dispos OR storage OR sharing OR shared OR expectation* OR request*)) (168,794)

	S37 MH “Drug-Seeking Behavior” (64)<br>S38 TX (compliance OR concordance OR adherence) (367,950)<br>S39 TX “Medication Adherence” (16,587)<br>S40 TX (“self-car*” OR "self-manag*" OR "self-monitor*" OR “self-medicat*”) (142,003)<br>S41 MH "Self Care" (43,627)<br>S42 MH "Self Medication" (2111)<br>S43 (S21 OR S22 OR S23 OR S24 OR S25 OR S26 OR S27 OR S28 OR S29 OR S30 OR S31 OR S32 OR S33 OR S34 OR S35 OR S36 OR S37 OR S38 OR S39 OR S40 OR S41) (1,735,029)<br>S44 S10 AND S19 AND S20 AND S42 (0)
**Number of references found**	0
**Electronic search report No. 5**	
**Electronic database**	APA PsycInfo <1806 to October Week 3 2021>
**Platform**	OVID
**Date of search**	27-04-2022
**Restriction of language**	None
**Other limits**	None
**Search strategy (results)**	1 (superbug* or superinfection*).ti,ab. 42<br>2 (multiresistant or multiresistance or "multi-resistant" or "multi-resistance" or MDR or MDRO or AMR).ti,ab. 405<br>3 ((drug* or multidrug or "multi-drug" or antimicrobial* or "anti-microbial*" or antiinfection* or "anti-infection*" or antibacterial* or "anti-bacterial*" or antibiotic* or "anti-biotic*") adj4 (resistant or resistance or tolerant or tolerance)).ti,ab. 4217<br>4 Drug Resistance.mh. 1990<br>5 Superinfection.mh. 9<br>6 "Drug Resistance, Multiple+".mh. 53<br>7 "Drug Resistance, Microbial+".mh. 61<br>8 Drug Tolerance.mh. 3554<br>9 ("r factor*" or "resistant factor*").ti,ab. 384<br>10 R Factors.mh. 2<br>11 ((antimicrobial or "anti-microbial" or antiinfection or "anti-infection" or antibacterial or "anti-bacterial" or antibiotic or "anti-biotic") adj4 stewardship).ti,ab. 86<br>12 Anti-Infective Agents.mh. 274<br>13 Anti-Bacterial Agents.mh. 1177<br>14 1 or 2 or 3 or 4 or 5 or 6 or 7 or 8 or 9 or 10 or 11 or 12 or 13 11308<br>15 (public or population or community or citizen* or household* or resident* or consumer* or carer* or caregiver* or people or adult* or men or women or mother* or father* or parent* or student* or pupil* or children or adolescent* or teenager*).ti,ab. 2531377<br>16 Caregivers.mh. 15137<br>17 Men.mh. 955<br>18 Women.mh. 3351<br>19 Parents.mh. 20783<br>20 Fathers.mh. 2733<br>21 Mothers.mh. 12531<br>22 Students.mh. 24851<br>23 15 or 16 or 17 or 18 or 19 or 20 or 21 or 22 2536114<br>24 intervention*.ti,ab. 432065

	25 (training or teaching or education or program* or initiative* or workshop* or seminar* or module* or session* or curriculum).ti,ab. 1078481<br>26 Teaching.mh. 8653<br>27 Teaching Materials.mh. 663<br>28 Education.mh. 3859<br>29 Models, Educational.mh. 1756<br>30 Health Education.mh. 9356<br>31 Education, Nonprofessional.mh. 286<br>32 Curriculum.mh. 9229<br>33 ((written or printed or online or oral or disseminat* or provi*) adj2 (information or advice or resource* or material*)).ti,ab. 66515<br>34 (pamphlet* or handout* or poster* or leaflet* or newsletter* or brochure* or booklet* or TV or television or radio or marketing or adverti?ing or publicity or campaign* or "mass media" or "communication media" or outreach or computer* or Internet or online or website* or "social media" or "social network*" or Facebook or Twitter or tweet or blog* or SMS or "short messaging service*" or text* or email* or podcast* or smartphone* or ((mobile or cell or smart) adj1 (phone* or telephone* or app* or application*))).ti,ab. 482949<br>35 Consumer Health Information.mh. 901<br>36 Health Promotion.mh. 14597<br>37 Health Communication.mh. 794<br>38 Persuasive Communication.mh. 1524<br>39 Public Health.mh. 4897<br>40 Audio Visual Aids.mh. 0<br>41 Manuals as Topic.mh. 1129<br>42 Pamphlets.mh. 640<br>43 Advertising as Topic.mh. 1922<br>44 Marketing.mh. 937<br>45 Social Marketing.mh. 875<br>46 Mass Media.mh. 2388<br>47 Social Networking.mh. 1175<br>48 Social Media.mh. 1851<br>49 (digital marketing or LinkedIn or Instagram or Pinterest or TikTok).ti,ab. 1520<br>50 24 or 25 or 26 or 27 or 28 or 29 or 30 or 31 or 32 or 33 or 34 or 35 or 36 or 37 or 38 or 39 or 40 or 41 or 42 or 43 or 44 or 45 or 46 or 47 or 48 or 49 1695593<br>51 Program Evaluation.mh. 13211<br>52 ((behavio#r adj3 (change* or changing or alter* or modification* or modify or modifying or modifies or modified)) or intention*).ti,ab. 90690<br>53 Motivation.mh. 29614<br>54 Intention.mh. 5740<br>55 (attitude* or belief* or view* or opinion* or expectation* or "anticipat* regret" or knowledge or awareness or "health literacy" or "risk perception*" or "perceived risk*" or "perceived susceptibility" or "illness perception*" or "treatment perception*").ti,ab. 1045231<br>56 Attitude to Health.mh. 25349<br>57 Health Knowledge, Attitudes, Practice.mh. 27538<br>58 Health Literacy.mh. 1639<br>59 ("perceived behavio#ral control" or autonomy or capacity or "self-efficacy" or competen* or confidence or ability or skill*).ti,ab. 732847<br>60 Self Efficacy.mh. 9898<br>61 ("perceived norm*" or "injunctive norm*" or "subjective norm*" or "descriptive norm*" or "moral norm*" or "self-identity").ti,ab. 9151<br>62 Social Norms.mh. 517

	63 ((medication* or drug* or antimicrobial* or "anti-microbial*" or antiinfection* or "anti-infection*" or antibacterial* or "anti-bacterial*" or antibiotic* or "anti-biotic*") adj4 ("use" or used or usage or consumption or purchas* or dispos* or storage or storing or sharing or shared or expect* or request*)).ti,ab. 71349<br>64 ((inappropriat* or irrational or imprudent or unnecessar* or irresponsibl* or misuse* or improper* or mistake* or indiscriminat* or suboptimum* or bad or overuse* or excessiv* or vary or varied or variation or poor or unsafe*) adj4 ("use" or usage or utili?e or treatment or consumption or purchas* or dispos* or storage or sharing or shared or expectation* or request*)).ti,ab. 20962<br>65 ((appropriat* or rational or prudent or judicious* or optimal* or correct or proper* or responsibl* or safe* or good or decreas* or limit* or curb* or minimi?e* or minimal* or lessen* or curtail* or abate* or restrict* or lower* or discontinue* or delay*) adj4 ("use" or usage or utili?e or treatment or consumption or purchas* or dispos or storage or sharing or shared or expectation* or request*)).ti,ab.86321<br>66 Drug-Seeking Behavior.mh. 649<br>67 (compliance or concordance or adherence).ti,ab. 62862<br>68 Medication Adherence.mh. 3851<br>69 ("self-car*" or "self-manag*" or "self-monitor*" or "self-medicat*").ti,ab. 29178<br>70 Self Care.mh. 7562<br>71 Self Medication.mh. 851<br>72 51 or 52 or 53 or 54 or 55 or 56 or 57 or 58 or 59 or 60 or 61 or 62 or 63 or 64 or 65 or 66 or 67 or 68 or 69 or 70 or 71 1817670<br>73 14 and 23 and 50 and 72 714<br>74 **(Reddit or Telegram).ti,ab. 279**<br>75 14 and 23 and 72 and 74 1
**Number of references found**	1

This one paper found was a dissertation which did not meet our study eligibility criteria.
