# Supplementary material for: Public Health Interventions to Improve Antimicrobial Resistance Awareness and Behavioural Change Associated with Antimicrobial Use: A Systematic Review Exploring the Use of Social Media
Source: Antibiotics (Basel). 2022 May 16;11(5):669. doi: 10.3390/antibiotics11050669 (PMC9137793; doi:10.3390/antibiotics11050669)
Supplement: Supplementary file 1 [file antibiotics-11-00669-s001.zip › File S3 - List of studies excluded.pdf]

File S3. List of studies excluded and reasons for their exclusion – full text

Nº	Title	Reason for exclusion
**1**	A School-Based Educational Intervention for School-Aged Children and Caregivers about Rational Use of Antibiotics in Urban Areas of Shaanxi Province: A Study Protocol for a Randomized Controlled Research.	It is a protocol, did not find the article with the results. Emailed author Correspondence: yufang@mail.xjtu.edu.cn but no response
**2**	Multicentre stepped-wedge cluster randomised controlled trial of an antimicrobial stewardship programme in residential aged care: protocol for the START trial.	The target audience included GPs and pharmacists
**3**	Comparing the effects of mobile-based education and booklet-based education on Iranian mothers' perception on antibiotics: a quasi-experimental study.	Participants recruited in healthcare setting
**4**	First Lebanese Antibiotic Awareness Week campaign: knowledge, attitudes and practices towards antibiotics.	The pre-test was for content comprehension of the questionnaire and modifications were made accordingly so that it was simple to understand and respond to. This study compared the measures only after the campaign.
**5**	Cost-benefit analysis of a population-based education program on the wise use of antibiotics.	It evaluates the cost
**7**	Antibiotic misuse of rural residents and pilot project of antibiotic take-back program	Conference presentation abstract
**11**	Preparing Parents to Make An Informed Choice About Antibiotic Use for Common Acute Respiratory Infections in Children: a Randomised Trial of Brief Decision Aids in a Hypothetical Scenario	Not at a public level, measuring at individual level
**12**	Antibio'Malin: An e-health resource to raise awareness of antibiotic stewardship and resistance in France	Part 1 evaluation by healthcare professionals and part 2 was mainly feedback. No apparent method clear.

**14**	Improving educator's knowledge and confidence to teach infection prevention and antimicrobial resistance	It's a train the trainer and not a population based intervention
**17**	Lesson learned from student-led one hundred days awareness on antimicrobial resistance in Nigeria	Conference presentation (abstract)
**18**	The efficacy of social cognitive theory-based self-care intervention for rational antibiotic use: a randomized trial.	Participants recruited in healthcare setting
**19**	The Mould that Changed the World: a quantitative and qualitative evaluation of knowledge and behavioural change in children in the UK following participation in a musical about antimicrobial resistance	Duplicate of paper 15
**20**	Reducing antibiotic self-medication practices in lagos southwest Nigeria: A community-based intervention study	Conference presentation (abstract)
**23**	Effect of public lecture using a skit to combat antimicrobial resistance	Article in Japanese
**26**	Public perceptions of antimicrobial resistance following the Keep Antibiotics Working national antimicrobial campaign	Conference presentation (abstract)
**28**	The "Obiettivo Antibiotico" Campaign on Prudent Use of Antibiotics in Sicily, Italy: The Pilot Phase.	This is a pilot study
**32**	Change in parental knowledge, attitudes and practice of antibiotic use after a national intervention programme.	The survey was conducted from 2014-2016 and participants were randomaly selected from public places and administred the survey. Based on the available information, we can assume its not the same paticipants that took the survey in 2014,2015 and 2016.<br>Health care professionals among the target audience
